# Psychosocial Aspects of the Lived Experience of Long COVID: A Systematic Review and Thematic Synthesis of Qualitative Studies

**DOI:** 10.1111/hex.70071

**Published:** 2024-10-24

**Authors:** Judith Eberhardt, Benjamin Gibson, Robert M. Portman, Nikki Carthy, Sam Rowlands, Rachel Batchelor, Laura Kane, Stephanie Kılınç

**Affiliations:** ^1^ School of Social Sciences, Humanities and Law Teesside University Middlesbrough UK; ^2^ School of Applied Social Sciences De Montfort University, The Gateway Leicester UK; ^3^ The Oxford Institute of Clinical Psychology Training and Research University of Oxford, Isis Education Centre, Warneford Hospital, Headington Oxford UK

**Keywords:** lived experience, long COVID, psychosocial, qualitative, thematic synthesis

## Abstract

**Background:**

Despite increasing recognition of long COVID, the psychosocial impacts of the lived experience on individuals remain underexplored. This systematic review sought to fill this gap by identifying key themes that describe the psychosocial dimensions of long COVID.

**Objective:**

The aim of this study is to identify key themes illustrating the psychosocial aspects of individuals' lived experience of long COVID.

**Search Strategy:**

Searches were conducted in multiple databases and grey literature sources for qualitative studies published between November 2019 and June 2024.

**Inclusion Criteria:**

Eligible studies involved adult participants self‐reporting long COVID. The studies needed to provide qualitative data that could be synthesised thematically.

**Data Extraction and Synthesis:**

Data extraction and thematic synthesis were conducted by at least two independent reviewers at each stage. Quality appraisal was performed using the Critical Appraisal Skills Programme tool.

**Results:**

The review included 34 studies. Thematic synthesis yielded five themes: ‘Debilitation’, ‘Uncertainty’, ‘Sources of Support’, ‘Meaning Making: Adjusting to a New Normal’ and ‘Experiences with Healthcare Services’. Individuals with long COVID reported experiencing physical, economic, and social challenges. Uncertainty and scepticism from others caused anxiety. Support from healthcare services, friends and online groups played an important role. Acceptance and gratitude were found to be meaningful in adjusting to the new normal. Experiences with healthcare services varied.

**Discussion and Conclusions:**

This review provides valuable insights into the psychosocial impact of long COVID, highlighting the profound changes and challenges that individuals face. Healthcare services should adopt a holistic approach to integrate psychosocial support within their management strategies, to improve overall patient outcomes.

## 1 Introduction

The general understanding of the aetiology of prolonged or fluctuating post‐COVID‐19 sequalae is limited, although several interacting mechanisms likely contribute [1, 2, 3]. ‘Long COVID’ [4] is a broadly agreed‐upon term used to refer to the experience of prolonged symptoms following infection with SARS‐CoV‐2 [5]. As it is broadly defined, long COVID, coined as such by those living with the condition [6], encapsulates previously defined ‘post‐COVID conditions’ [7, 8] as well as ‘ongoing symptomatic COVID‐19’ and ‘post COVID‐19 syndrome’ [9]. Common physical symptoms include fatigue, breathlessness, coughs, chest pains, post‐exertional malaise, cognitive difficulties (including concentration impairment and ‘brain fog’), headaches, light‐headedness, sleep problems, musculoskeletal pain, chest pain and other health problems [10, 11, 12, 13].

Nevertheless, a recent systematic review found that the second most common characteristic of long COVID was not, in fact, a symptom, but poor quality of life (QoL) [14]. Given the types of symptoms associated with long COVID, it is perhaps not surprising that people's QoL suffers. However, it has been argued that long COVID's impact on people's lives is overlooked and under‐researched [15]. Of 37 studies reviewed as part of a review of long COVID symptoms [1], only four reported on the associated psychosocial impacts. Specifically, studies reported findings of comorbid posttraumatic stress disorder, depression and anxiety, increased requirements for personal care and limiting of activities undertaken as part of daily living [16, 17, 18, 19]. To date, research has focused more on the prevalence and types of symptoms rather than how these symptoms may impact on people's lives [14, 20]. This is despite the fact that persistent symptoms may contribute to larger health issues, which may, for example, affect people's social relationships and their ability to meaningfully engage with work and any type of activity involving even basic physical activity [15].

A qualitative, phenomenological investigation into the psychosocial impacts associated with the condition [21] found that people with long COVID reported a total change of life due to symptomatology, including a loss of autonomy that affected their social, family and professional lives. Individuals with long COVID reported experiencing feelings of social isolation, a sense of abandonment that was often amplified by stigma and difficulties being believed and receiving a diagnosis. There were also issues with managing symptoms and accessing care services, as well as a sense of living with uncertainty that individuals with long COVID felt was caused by a general lack of institutional, social, professional, familial and medical support.

Structural inequalities such as financial constraints, lack of resources, overcrowding, poor working conditions and inability to access services were important factors in the spread of COVID‐19 and evidence suggests that they may similarly form an important context for long COVID [22, 23, 24]. Furthermore, loss of work, income and social interaction associated with being chronically ill, with unpredictable relapses and periods of sickness, will likely have negative impacts on mental health, which can in turn lead to further loss of work, income and social interaction, culminating in social exclusion and a vicious circle [25].

Examining the lived experience of a phenomenon such as long COVID places participants at the centre of the illness experience [26], recognising them as experts on their condition [27]. Drawing on the critical knowledge of those living with long COVID allows the key issues affecting this group to be explored and used to inform and improve support services. Given that the psychosocial impact of living with long COVID in particular has received such poor attention [28], which arguably should not be overlooked in favour of purely medical and rehabilitation approaches to symptoms [29], this systematic review aimed to synthesise current evidence to identify and explore key themes illustrating the psychosocial aspects of the lived experience of long COVID. The review aimed to look beyond the impact of long COVID symptoms and experiences of healthcare, to the broader psychosocial impact of living with long COVID. The review question was as follows: What are the psychosocial aspects of the lived experience of long COVID?

## 2 Methods

### 2.1 Search Strategy

This systematic review protocol was preregistered through the International Prospective Register of Systematic Reviews (PROSPERO CRD42022343091); see Appendix S1 for the protocol. The Preferred Reporting Items for Systematic Reviews (PRISMA) guidelines [30] were followed in the reporting of this review. The SPIDER (Sample, Phenomenon of Interest, Design, Evaluation, Research type) search tool for qualitative systematic reviews was chosen as a reliable method of defining the research question and search strategy for qualitative reviews [31]. Tables 1 and 2 show how the SPIDER search tool informed the search terms.

**Table 1:** Use of SPIDER to define research question and search criteria.

*S*ample	People living with long COVID
*P*henomenon of *I*nterest	Psychosocial aspects of living with long COVID
*D*esign	Any design that uses participant quotes
*E*valuation	Studies related to the lived experience of people with long COVID, excluding lived experience of their family or carers
*R*esearch type	Qualitative, including mixed methods studies that contain sufficient qualitative data, excluding qualitative evaluations of specific interventions

**Table 2:** Search terms.

Sample	Phenomenon of Interest	Design	Evaluation	Research type
Long COVID Longer term effects of COVID‐19 Post acute sequelae of COVID‐19 (PASC) Post COVID‐19 syndrome Post COVID‐19 condition Chronic COVID syndrome (CCS) Long‐haul COVID	Psychosocial Psychol* Social	Phenomenol* Interpret* Narrat* Story Stories Theme* Thematic Case stud* Interview Focus group Grounded theory	Lived experience Living with Life with Personal Voice Meaning	Qualitative Mixed methods

The search was conducted on PsycINFO, MEDLINE, AMED, APA, CINAHL and the Psychology and Behavioural Sciences collection. Grey literature was also searched via Google, inputting the search terms and examining the first 10 pages of the search results, focusing on primary research studies and long COVID relevant organisation reports (e.g., NHS, Health Foundation, Kings Fund, NIHR, Royal Society, Tony Blair Institute for Global Change).

Articles published between November 2019 and June 2024 were searched. The first search was performed in June 2022, and the search was then updated in February 2023, and again in June 2024. (an example of the Medline search can be found in Table S1.)

### 2.2 Eligibility Criteria

Articles and reports were eligible for inclusion in the review if they reported primary research, focused on long COVID, adopted a qualitative approach, data were gathered from adults who self‐reported as living with long COVID and the focus was on their lived experience of long COVID. The decision was made not to use a recognised definition of long COVID, as the definition changed in the early months as we learnt more about long COVID. If the sample included non‐long COVID sufferers, the data were included only if it was clearly attributable to long COVID sufferers. Mixed methods studies that contained sufficient qualitative data were also included in the review, as were papers and reports from any country that were translated into English.

**Figure 1:**
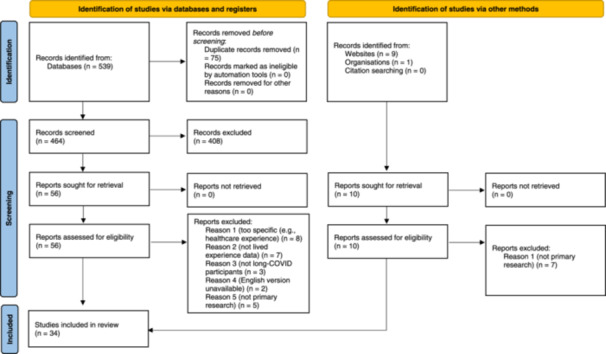
PRISMA flow diagram.

Articles were excluded if they were service evaluations, qualitative evaluations of specific interventions, papers or reports that did not report primary research, editorials, magazine articles, books, dissertations/theses, conference abstracts and posters. Literature not reporting first‐person experiences of long COVID was also excluded, as well as quantitative studies, articles or reports where the full text could not be retrieved and papers and reports focusing on children with long COVID (see Figure 1 for PRISMA flowchart).

### 2.3 Selection Process

To ensure rigour during data extraction, four reviewers took part in the process. The first reviewer (S.K.) conducted the searches and exported the search results into Zotero where duplicates were removed. S.K. screened all papers and reports by title and abstract and the second reviewer (S.R.) independently 20% by following the inclusion and exclusion criteria. There were no discrepancies between S.K. and S.R. during this process. S.K. then retrieved the full texts of the selected papers and reports. All full‐text papers and reports were screened for relevance by both S.K. and the third reviewer (J.E.) independently. Consensus over inclusion was reached via a meeting between both reviewers. The same process was completed for grey literature by J.E. and a fourth reviewer (B.G.).

As part of data extraction, a study characteristics table was completed by the fifth reviewer (N.C.), which detailed the title, author, year published, country of origin, study aim, method of data collection, method of data analysis, study population characteristics and key findings or themes. An inclusive approach to data extraction was utilised [32]. N.C. and the sixth reviewer (R.B.) both independently appraised the quality of the final studies using the Critical Appraisal Skills Programme (CASP) tool [33], with J.E. reviewing 50% of each reviewer's final checklists to ensure consistency. Any disagreement in CASP ratings was discussed in a meeting between reviewers and a consensus was reached. No study was excluded as a result of using the CASP tool, though it was used to provide context to the review.

### 2.4 Thematic Synthesis

The qualitative studies included in the analysis were examined using thematic synthesis, a systematic approach that involved coding extracted quotations line by line and developing descriptive and analytical themes [34]. Quotes from participants living with long COVID were recorded in a Microsoft Excel spreadsheet, along with their corresponding themes and sub‐themes from the original studies. These quotes were then compiled into a study transcript in a Microsoft Word document, omitting the accompanying theme details. One author (R.M.P.) coded the quotations on the basis of their meaning and context. Another author (J.E.) independently reviewed the coding for agreement and provided suggestions for reorganising themes and sub‐themes. The study team reached a consensus on the final set of themes and sub‐themes. Finally, the study team developed interpretations beyond the original content of the studies, offering a comprehensive overview of the lived experience of long COVID. To maintain reflexivity as much as possible, the authors considered the data and their interpretations for competing conclusions throughout.

## 3 Results

### 3.1 Study Characteristics

The search strategy returned 539 articles (10 of these grey literature), all of which were reviewed by abstract and title. This resulted in 66 articles being subject to full‐text review (7 of these grey literature), of which 34 (3 of these grey literature) were included in the final review [35, 36, 37, 38, 39, 40, 41, 42, 43, 44, 45, 46, 47, 48, 49, 50, 51, 52, 53, 54, 55, 56, 57, 58, 59, 60, 61, 62, 63, 64, 65, 66, 67, 68]. Of these, 18 were conducted in the United Kingdom (UK), 8 in the USA, 2 in Italy, 1 in Spain, 1 in Denmark, 1 in China, 1 in Canada and 2 across several countries (Canada/Ireland/UK/USA and Canada/UK/USA, respectively). A total of 1493 participants took part; in addition, there were a total of 92 responses in text message or other formats. Twenty‐seven studies had mostly female participants, two had female participants only, one had male participants only, one study had equal numbers of male and female participants and four did not report gender distribution. Of those studies that reported ethnicity, most had predominantly White participants, and one had only Black participants. One paper included healthcare professionals with long COVID in their samples [44], and two papers focused only on healthcare professionals [45, 56]. Not all studies reported information on duration of symptoms; among those that did offer this information, duration ranged from 3 weeks to 24 months. A variety of data collection methods was used; however, most studies used semi‐structured interviews or focus groups. Most studies (26) used some form of thematic analysis; one used a phenomenological–hermeneutic approach, one used content analysis, two used inductive thematic content analysis, one used grounded theory and three studies did not report the method of analysis used. Twenty‐four of the studies were assessed to be of high quality, nine of moderate quality and one of low quality (see Table S2 for study characteristics).

### 3.2 Synthesis of Extracted Findings

The thematic synthesis of extracted findings yielded the creation of five superordinate themes (see Table 3).

**Table 3:** Superordinate themes and sub‐themes.

Themes and sub‐themes	Illustrative quotes
Theme 1: Debilitation	
Sub‐theme: The physical impact of long COVID	*So if I do something physical I suffer. If I walk I suffer in my legs. If I do something with my hands I suffer with my hands. If I start to think too much I then get a foggy head. If I type an email on the computer and it goes on too long, I then can't think enough to shut the computer down* [41].
Sub‐theme: The economic impact of long COVID	*I go to the doctor a lot, right? I have a lot of copays. I have a lot of tests that they run me through. I do have several thousand dollars of medical bills that I no longer had before. There is that side effect, or that burden. If that's as bad as it gets economically, at least I still have a job. I still have insurance and stuff* [63].
Sub‐theme: The social impact of long COVID	*Relationships with friends are strained as my energy levels mean I am not able to meet up or keep in touch as readily as I would like* [38]. *I've changed a lot as a person. So earlier, it was go up the career ladder, follow this thing, be on all the time, deliver, get that next promotion. And that was my life, it has always been that way. And this [Long COVID] has made me think what is more important in my life, and how not to take health for granted* [66].
Theme 2: Uncertainty	*I can't live like this, how am I going to go on in the future, am I going to get worse, am I going to get better? Obviously, you think of everything. How are you going to look after your kids, the family, your work situation, how are you going to pay your bills* [35]. *Some days, it's so scary, like I don't know if I'll ever, ever feel the same* [59]. *The scary thing about Long Covid is it keeps developing. For me it was like ping pong all over my body, one minute it's in my chest, one minute it's in my muscles, one minute it's in my gut… it just keeps moving around the body and sometimes it's better and sometimes it's worse* [62].
Sub‐theme—Scepticism from others	*My daughter, for example, does not believe in COVID and my friends are not vaccinated and do not believe in it. I tell them that I have long COVID but they tell me that I'm making things up in my head, and that makes you feel alone* [53].
Theme 3: Sources of support	*At one point, I'm like, “What the hell is wrong with me? I don't understand.” I started to search online and I found people on Reddit. I was like, oh, okay, this is a thing. Like, I'm not crazy … It gave me information … It validated [my symptoms]* [52]. *A lot of [my anxiety] was feeling like, I don't know how long my symptoms are going to last. I'm feeling like I don't know anybody else who's experiencing similar symptoms … I started looking at the COVID Facebook posts and connecting with people who have similar symptoms … And I felt like that night, after I joined that COVID Facebook group, was the first night in a very long time that I didn't have that massive anxiety attack. It's definitely been very helpful to connect with people who have similar symptoms, especially when a lot is unknown about the virus … I just felt like it opened up a whole new channel of hope and support and resources* [52]. *So the Facebook community…that community was was amazing because you knew for the first time that it wasn't just you and it wasn't. You weren't alone. So that was incredible* [67].
Theme 4: Meaning making: adjusting to a new normal	*So as much as I'm enjoying [walking the dog], it has the knock‐on effect. But that is getting less and less, so the more I'm doing the better I'm feeling afterwards. I think [relapses are] all part of it, just got to get on with it and push myself a little bit harder and then hopefully I'll get better quicker. It doesn't put me off* [41].
Theme 5: Experiences with healthcare services	*I've spoken to four different GPs throughout this. I've not found them very helpful, so to be honest at all* [43]. *I felt dismissed by my doctors and started feeling like I was a nuisance because I kept pestering them and they did not believe me. I felt like no one around me understood. I had support around me. My family and friends were supportive, but I still felt that they did not understand, and I felt so alone* [65].
Sub‐theme: Recommendations for improving the patient experience	*My expectation of such a clinic would be to rule out treatable causes or complications, based on our symptoms. And then active involvement with physiotherapies and occupational therapies maybe a psychologist […] we now know that COVID is a multi‐system disease so the fact that you don't display signs of respiratory infection doesn't mean that you don't have a problem* [45].

#### 3.2.1 Theme 1: Debilitation

‘Debilitation’ was noted in 15 studies [35, 37, 38, 40, 41, 46, 47, 48, 50, 57, 58, 62, 63, 66, 68], documenting the myriad negative changes that participants experienced attributed to long COVID. This theme centred around the conceit that participants were either unable to do things at the same level as they had before contracting long COVID or simply unable to do them at all:

The slightest thing was an effort in a way I've never ever conceived before, it's the most fatigued I have ever been … things like changing my bedding, I did in stages like one pillowcase and then later in the day I'd do another pillowcase, it was that sort of level of difficulty with day‐to‐day tasks *[* *41* *]*.

I used to be a happy girl who wanted it all (begins to cry) I still want it all, but uh…but I cannot…Well, I think it has been hard to [make] heads or tails of all this. There have been so many adjustments…and ways I've had to reinvent myself…I somehow became more and more introverted…Uhm, people said that there was something different about me…I have a colleague that said I seem a bit melancholic (still crying) *[* *46* *]*.

Consequently, participants in some studies expressed a sense of grieving for the person they used to be:

It feels like Long Covid has taken all the joy out of my life. I am trying to adapt and find new interests and hobbies, but I am not able to be the person I was before. It has robbed me of my social life, my independence, my purpose, my career, my creativity *[* *58* *]*.

We constructed three sub‐ordinate sub‐themes specifically relating to the physical, economic and social debilitation experienced by participants. These themes are presented as distinct sub‐themes but are intrinsically interwoven within the superordinate theme of debilitation, as well as overlapping with one another.

##### 3.2.1.1 Sub‐theme: The Physical Impact of Long COVID

The physical impact of long COVID was evident in eight studies [35, 38, 40, 42, 47, 57, 62, 68]. Participants highlighted a negative change in their physical capacity and capabilities following the onset of long COVID, describing how tasks that would typically (and previously) require minimal energy expenditure now required a seemingly disproportionate amount of effort to achieve:

I couldn't have even picked the laptop up and opened it, to be honest, you're completely just wiped out *[* *35* *]*.

[I have] frustration at being unable to function as well as I did before; the projects I am involved with take so much longer, and need double‐ and triple‐checking to ensure no stupid errors *[* *38* *]*.

##### 3.2.1.2 Sub‐theme: The Economic Impact of long‐COVID

Participants referenced the economic impact of long COVID in eight studies [37, 40, 41, 46, 47, 50, 63, 68]. They described how long COVID had negatively impacted on their ability to perform work‐related tasks and responsibilities. This created anxiety regarding whether participants could maintain employment, and thus, maintain their level of financial income. As previously, this was represented as an inability to perform necessary tasks to the same levels as previously achieved, or simply being unable to perform the tasks at all:

I have had to cut my working hours in half as it is too exhausting working full time. […] I don't think I will be able to keep my job. I'm barely getting by doing the minimum *[* *57* *]*.

In some instances, participants described how their workplace had been able to make reasonable adjustments to support them:

There was too much noise and [too many] customers calling in. I did not grasp a piece of what they were saying. It was just a big mess for me. Plus, my boss just kept throwing tasks at me, and I kept saying, ‘I can't handle anymore. You can't keep [sending] me tasks all the time’. I ended up on sick leave…He (the boss) called me after a few days and asked how he could help me…We agreed that I move to another location…where no one came running and that I had no phone calls…This worked much better because I could take my breaks…It was so much better to sit there alone. I think I sat there [at the new location] for a month or two *[* *46* *]*.

##### 3.2.1.3 Sub‐theme: The Social Impact of Long‐COVID

The detrimental impact of long COVID on participants' social lives was present in seven studies [35, 37, 46, 47, 58, 66, 68]. Participants frequently referenced a diminished capacity to engage in social interaction with friends, explaining how ‘normal’ social activities were now perceived to be beyond their capability:

Most people maybe get together and have a pizza in the evening… from 6 p.m. onwards I start my fever and I literally fall asleep between 9 and 10 p.m. Now it becomes really difficult for me to sustain an evening in the company, also because I can't even stand the confusion, the noise […] So in the very normal social relationships I had before, I'm scaling everything down according to my strengths, my possibilities, when I succeed *[* *47* *]*.

Typically, this contributed to reduced social contact and thus, the deterioration of previously held friendships:

Relationships with friends are strained as my energy levels mean I am not able to meet up or keep in touch as readily as I would like *[* *38* *]*.

#### 3.2.2 Theme 2: Uncertainty

‘Uncertainty’ was present in 14 studies [35, 43, 45, 47, 50, 51, 52, 53, 55, 57, 59, 68], reflecting participants' and healthcare professionals' initial—and sometimes continuing—lack of understanding regarding what long COVID is, and the relative ambiguity regarding what could be considered ‘normal’ physical and psychological consequences of long COVID. Unsurprisingly, this uncertainty led to feelings of anxiety among participants:

For the first 9 months of long COVID, the fact that nobody knew anything about long COVID, how to treat it and whether it would be chronic increased a sense of hopelessness which definitely affected my mental health and quality of life *[* *57* *]*.

This uncertainty was also characterised by participants in some studies experiencing cyclical waves of recovery and relapse; individuals would feel as though their health was improving, only to see it suddenly deteriorate again in an unpredictable manner:

*There's a lot of relapses involved … which, initially, was very, very demoralising, because you think, or I would think, I've turned a corner, I'm going to get better, and then it would just suddenly get, like take two steps forward and three or four steps back. And that's characterised the entire journey I've had with COVID, up until now really* [35].

##### 3.2.2.1 Sub‐Theme: Scepticism From Others

Within the superordinate theme of uncertainty, we identified a sub‐theme—scepticism from others—specifically related to participants' experiences of others seemingly not believing in the existence of long COVID. Scepticism from others was noted in seven studies [35, 45, 47, 49, 53, 54, 57]. Participants recounted their frustrations when their experiences were directly challenged, or the severity of long COVID was seemingly diminished, by healthcare providers, as well as friends and family:

A lot of people don't understand long COVID, so when you explain to them, I'm still not feeling right 6 months down the line, a lot of people have said, I think you're just worrying too much. That's what I think my parents come back with. They keep saying to me, you worry too much, there's nothing wrong with you *[* *35* *]*.

My daughter, for example, does not believe in COVID and my friends are not vaccinated and do not believe in it. I tell them that I have long COVID but they tell me that I'm making things up in my head, and that makes you feel alone *[* *53* *]*.

#### 3.2.3 Theme 3: Sources of Support

This theme synthesises participants' varying support experiences throughout their long COVID journeys and was noted in 11 studies [35, 40, 42, 45, 49, 51, 52, 53, 57, 60, 67]. Support encompassed various sources, including healthcare services, friends and family, and online support services, and participants reported varying perceived levels of support in relation to the support received from each of these sources. The most consistent finding was in relation to the prominence of long COVID support groups for providing companionship and reassurance:

It's a relief to me that everything I've gone through in the last eight months is finally being validated by other people now having very similar experiences as mine post‐covid. I've felt very alone in this whole experience to this point *[* *42* *]*.

I'm extremely grateful to the support groups online and those people who've shared their stories already *[* *49* *]*.

#### 3.2.4 Theme 4: Meaning Making: Adjusting to a New Normal

Building upon previous themes, particularly ‘Debilitation’ and ‘Uncertainty’, participants in six studies [35, 41, 51, 54, 56, 68] reported on the ways in which they were realigning their perspectives in accordance with their new status as an individual living with long COVID. Participants spoke of the importance of acceptance, positivity and gratitude, as highlighted in the following quotes, respectively:

I'm self‐aware when it comes to my health and my mood and I think I'm just accepting of it rather than wanting to change it or be negative about it *[* *41* *]*.

Even though life is hard now, I have to face the future positively! Being healthy is always the most important thing! To try all the things that I was afraid to try before! *[* *56* *]*

As evidenced in the last extract, this sense of adjusting to a new normal also manifested behavioural adaptations alongside these psychological adaptations.

#### 3.2.5 Theme 5: Experiences With Healthcare Services

Participants' experiences with healthcare services were detailed in 11 studies [35, 38, 40, 42, 43, 44, 47, 49, 50, 65, 67]. There was some evidence of positive experiences:

In the early days, my GP was fantastic … I sent him a letter to tell him what was going on in my life, all my symptoms and everything. And I said, I am so sorry about harassing you. And he phoned me up, and he said, keep harassing me, he said, if you don't tell me, I don't know *[* *35* *]*.

However, most participants, in most of the included studies, remarked upon negative experiences with healthcare services. Mostly, this revolved around healthcare services being unable to provide any resolution regarding the identification of a cause(s) for the health issues that participants were experiencing:

It has felt like there was no understanding from the Doctors. I was having to send medical evidence, pictures etc. to them, for them to listen to me. My Doctors certificate says ‘numerous unexplained conditions’ how can this be? *[* *42* *]*

Participants' dissatisfaction also extended to include healthcare services that seemingly downplayed the severity of their health problems:

They said “ok we'll get someone to phone you”. My GP called back and just said “oh well it's probably anxiety”. He didn't seem to have any idea what it could be. I felt fobbed off. I said I'm worried – there are articles and news outlets that I've been reading and I want to know what's happening to me – people are having strokes, blood clots. I haven't been to hospital but I'm concerned I'm still getting these effects. He said “oh you'll be fine you've only had it mildly” *[* *44* *]*.

##### 3.2.5.1 Sub‐Theme: Recommendations for Improving the Patient Experience

Five studies [38, 40, 43, 45, 50] included participant‐led recommendations for how the long COVID patient experience could be improved. Predominantly, these recommendations focussed on providing reassurance that the symptoms would not be detrimental to participants' long‐term health via follow‐up checks:

I think probably what would be good would be some sort of just having my body checked to reassure me that there's no long term damage or some monitoring to, because we don't know what the virus can do to your body. I suppose the things that would reassure me are things where I need my body actually checking which I don't think you could check online, you can't check for blood clots online, you can't check for neurological damage online can you? *[* *43* *]*

## 4 Discussion

The findings from this systematic review provide valuable insights into the lived experiences of individuals with long COVID. The five superordinate themes that were derived—Debilitation, Uncertainty, Sources of Support, Meaning Making: Adjusting to a New Normal and Experiences with Healthcare Services—highlight the multifaceted impact of long COVID on individuals' lives.

Individuals experienced profound negative changes attributed to long COVID. They described a range of challenges, from diminished physical capacity to significant disruptions in their social and economic lives. The physical impact of long COVID was evident in participants' accounts, with tasks that were once effortless now requiring substantial effort. This physical debilitation had negative effects on their ability to work and maintain financial stability. The economic impact of long COVID was characterised by participants' reduced work hours, struggles to perform tasks and concerns about job security. The physical and economic impact of living with long COVID has also been documented in the literature (e.g., [69, 70]). In addition, the social impact of long COVID was reflected in individuals' diminished social interactions, strained relationships and a sense of isolation. This is in line with prior literature [71, 72, 73].

The findings around uncertainty highlight the lack of understanding and ambiguity surrounding long COVID. This uncertainty created anxiety and feelings of hopelessness among participants, as they grappled with the unknown long‐term consequences of the condition. The cyclical nature of recovery and relapse further added to the uncertainty, leaving participants feeling demoralised and unsure about their prospects for improvement. Moreover, participants reported experiencing scepticism from others, including healthcare providers, friends and family, who doubted the existence or severity of their long COVID symptoms, further compounding individuals' struggles and making them feel isolated. The fluctuating, episodic nature of long COVID is supported by other research highlighting long COVID as an illness characterised by health‐related challenges or disability that may be multidimensional and unpredictable in nature [74]. Recognising the impact of this unpredictability on individuals therefore needs to be part of the management of long COVID.

In the studies reviewed here, various sources of social support were drawn upon by individuals. Although the experiences varied, support groups, both online and in person, were identified as valuable sources of companionship and reassurance for many participants. These support groups provided validation, a sense of belonging and the opportunity to connect with others who shared similar experiences. Healthcare services, friends and family also played important roles in participants' support systems, although the perceived level of support varied across individuals and contexts. The importance of social support in living with long COVID has been documented in prior research [75, 76, 77]. Our findings highlight the central role of social support, or lack thereof, in the lived experience of long COVID. However, the present review also illustrates the mixed experiences of patients with healthcare professionals, with some praising the support and understanding received from healthcare providers and others expressing dissatisfaction as a result of challenges in obtaining medical validation, being dismissed or misunderstood and feeling fobbed off. Recommendations for improving the patient experience were provided by participants, such as follow‐up checks and reassurance regarding long‐term health outcomes.

The present findings also draw attention to efforts to make sense of a new reality and adapt to living with long COVID. Reviewed studies emphasised the importance of acceptance, positivity and gratitude in navigating one's changed circumstances. This process of adjustment involved not only psychological adaptations but also behavioural changes as participants sought to align their perspectives and actions with their new status as individuals living with long COVID. The importance of humility and acceptance when working within one's limits with long COVID has been previously discussed [78].

Overall, our findings suggest that long COVID has a significant impact on individuals' lives, encompassing not just physical but also psychosocial and economic dimensions. The experiences reported highlight the need for increased awareness, understanding and support for those living with long COVID. Healthcare professionals play a crucial role in addressing the uncertainties and challenges faced by individuals with long COVID, and improvements in healthcare services are necessary to provide adequate support and validation. In addition, the value of peer support through support groups, both online and in person, should be acknowledged and promoted. A holistic, comprehensive approach [79] using a biopsychosocial model [80] is recommended to better capture the interplay between biological, psychological and social factors in understanding and managing long COVID. This approach recognises the importance of considering not only the physical symptoms but also the psychological well‐being and social support systems of individuals. By recognising and addressing the multifaceted experiences of individuals with long COVID, healthcare systems and society can work towards better supporting and improving the QoL for those affected by long COVID.

## 5 Strengths and Limitations

Limitations of the present review need to be acknowledged. One concerns the self‐report nature and varying inclusion criteria used among the studies analysed, which is at least in part a result of inconsistent and evolving definitions of long COVID across studies. It is important for future studies to adopt a standardised definition of long COVID and a verified diagnosis of the illness, to facilitate comparability and a deeper understanding of the psychosocial aspects of the lived experience of the illness as knowledge continues to evolve.

Sampling bias may have impacted on the findings of this review, as the participants who chose to take part in the reviewed studies may have felt more motivated to share their experiences due to feeling particularly impacted by long COVID. In addition, the majority of included studies were conducted in Western countries and limiting the search to studies published in the English language meant that studies in different cultural contexts may have also been missed. Thus, different healthcare services and provisions may have impacted the presented findings and the results of the review may not fully reflect the experiences of participants from different geographical locations or cultural backgrounds.

There was a lack of detailed demographic data in the included studies, raising concerns about the representation of underrepresented, socioeconomically deprived and minority groups. These populations often bear a disproportionate disease burden; yet, their experiences may be underrepresented in the findings. This reflects a broader issue in long COVID research. Future studies should prioritise including these groups to ensure that interventions are equitable and address health inequalities effectively.

Another limitation relates to the varying methodological rigour observed in the included studies, although it needs to be pointed out that 24 of the 34 studies were rated to be of high quality and nine of moderate quality. Furthermore, the interpretation of data in qualitative studies can be subjective. To mitigate this limitation, a rigorous approach was used in this review. A minimum of two independent reviewers were involved at every stage of the review process, to enhance the reliability and rigour of the findings.

The themes identified and discussed in this review broadly generalise the psychosocial impacts of long COVID across the study population. However, long COVID affects individuals differently, potentially influenced by the severity of their initial infection, whether they had confirmed clinical testing or repeat infections and pre‐existing health conditions. Furthermore, there may be demographic variations in individuals' experiences. These factors could stratify the long COVID population into subgroups with distinct experiences and needs. This was beyond the scope of the present review, which primarily aimed to capture a wide spectrum of experiences to provide a comprehensive overview of the condition's effects, rather than examining specific subgroup differences. Future research should aim to stratify participants on the basis of clinical characteristics and demographic factors to better understand the varied impacts within distinct groups. Furthermore, three of the studies included in this review featured healthcare professionals with long COVID. These studies were included, as they contribute to the broader understanding of long COVID's psychosocial impact. Future research could examine the specific challenges faced by professionals related to their dual roles as patients and providers, which may influence their experiences and coping strategies.

This review integrates qualitative studies with distinct methodological paradigms and ontological assumptions, which presents challenges in ensuring complete commensurability across findings. Despite this, the diversity of perspectives helps provide a comprehensive understanding of long COVID experiences, although it limits the uniform interpretation and integration of themes, which must be considered when evaluating the findings.

A notable strength of the present review is the inclusion of grey literature alongside peer‐reviewed publications, contributing to a more comprehensive analysis by incorporating a broader range of sources, thus increasing the inclusivity of the findings. An additional strength lies in its focus on the psychosocial aspects of the lived experience of long COVID. By emphasising the psychological, social and emotional impacts of the condition, this review sheds light on an important dimension requiring better understanding to effectively support individuals affected by long COVID.

## 6 Conclusion

Long COVID can have a profound impact on individuals, pervading aspects of life such as one's experience of their own body, identity, personal and social identity, relationships and one's feeling of agency over one's own life. Living with long COVID is a challenging and multifaceted experience that requires a holistic approach. By further investigating the unique aspects of this condition and developing tailored interventions, we can improve the care, support and overall well‐being of individuals living with long COVID.

## Author Contributions

**Judith Eberhardt:** conceptualisation; methodology; validation; writing–original draft; formal analysis; writing–review and editing; investigation. **Benjamin Gibson:** writing–original draft; formal analysis. **Robert M. Portman:** formal analysis; writing–original draft; validation. **Nikki Carthy:** formal analysis; validation. **Sam Rowlands:** formal analysis; validation; writing–review and editing. **Rachel Batchelor:** writing–review and editing; formal analysis; validation. **Laura Kane:** formal analysis; validation; investigation. **Stephanie Kılınç:** methodology; conceptualisation; formal analysis; validation; writing–review and editing; investigation.

## Ethics Statement

With this study being a systematic review, there was no direct patient or public involvement. The articles reviewed were selected on the basis of their relevance and contribution to understanding the psychosocial aspects of the lived experience of long COVID, and all data analysed were derived from these existing sources. However, some of the articles included in this review have had patient and/or public input.

## Conflicts of Interest

The authors declare no conflicts of interest.

## Supporting information

Supporting information.

## Data Availability

With this being a systematic review, no new data were generated for this study.
