# Supplementary material for: Psychosocial Aspects of the Lived Experience of Long COVID: A Systematic Review and Thematic Synthesis of Qualitative Studies
Source: Health Expect. 2024 Oct 24;27(5):e70071. doi: 10.1111/hex.70071 (PMC11500211; doi:10.1111/hex.70071)
Supplement: Supplementary file 1 — Supporting information. [file HEX-27-e70071-s001.docx]

Table S1. Example MEDLINE search

**Limiters: human participants**

**Year: 2019 - 2024**

**Population = 18 years+**

S1: Long-covid OR Longer-term effects of covid-19 OR Post acute sequelae of covid-19 (PASC) OR Post covid-19 syndrome OR Post covid-19 condition OR Chronic covid syndrome (CCS) OR Long haul covid

S2: Psychosocial OR Psychol* OR Social)

S3: Phenomenol* OR Interpret* OR Narrat* OR Story OR Stories OR Theme* OR Thematic OR Case stud* OR Interview OR Focus group OR Grounded theory

S4: Lived experience OR Living with OR Life with OR Personal OR Voice OR Meaning

S5: Qualitative OR Mixed methods

S3 OR S4 OR S5 = S6

S1 AND S2 AND S6

| Qualitative* | AND neurological* | AND phenomeno*  OR living with  OR lived experience  OR experience/s  OR perspective  OR patient reported*  OR story  OR stories  OR narrative*  OR life with |
| --- | --- | --- |

**Table S2. Study characteristics**

| **Study (Author/ year)** | **Country** | **Population** | **Minimum and/or average duration of long-COVID symptoms** | **Issue** | **Outcome** | **Method** | **Analysis** | **Findings** | **CASP Quality appraisal** |
| --- | --- | --- | --- | --- | --- | --- | --- | --- | --- |
| Aghaei et al. (2022) | USA | 15 women, aged between 26 and 35 years | Participants were required to have been infected with COVID-19 and having experienced at least one COVID-19 symptom lasting four weeks or longer after a COVID-19 diagnosis. Minimum/average duration not stated. | The effects of long COVID on the social life of female long haulers, exploring the effects of long COVID physical and psychological  symptoms on various aspects of women’s social life  . | A conceptual model that illustrates the impacts of long-COVID on social aspects of female long haulers’ life.  treatment process and patient role,  perceived body image, and stigma | Semi-structured online interviews | Thematic analysis | Physical issues:  Limited physical activities, altered perceived body image  Economic issues: job insecurity, financial hardship  Social relationships:  Social isolation, social reactions to long haulers symptoms. Changed communication methods, decreased social capital  Conflicts with social roles: job roles, family roles, patient role.  Social stigma:  Stigma labels, stigma outcomes | Moderate |
| Bergmans et al. (2023) | USA | 15 Black American adults with Long COVID; 80% were female. | Participants had to report physical or mental health symptoms that lingered over 1 month after an acute COVID infection. Minimum/average duration not reported. | This study aimed to examine the impact of long COVID on quality of life and symptom management approaches among Black American adults. | Using an interpretive description design, this study helps address  the underrepresentation of Black American perspectives and experiences  in long COVID research. Our findings revealed how long  COVID affects personal identity and physical functioning among  Black adults, as well as the range of strategies that participants  used to manage their symptoms, including self-directed  lifestyle  changes, leaning on social networks, and positive thinking.  However, social disadvantage interfered with symptom management. | Race-concordant semi-structured interviews | Inductive Thematic Analysis | Four themes: (1) The impact of long COVID symptoms on personal  identity and pre-existing  conditions; (2) Self-management  strategies for long  COVID symptoms; (3) Social determinants of health and symptom management; and  (4) Effects on interpersonal relationships. | High |
| Burton et al. (2022) | UK | 21 people with long-COVID participated in the study. Sample was predominantly female (67%) and White British (67%) | Participants must have been experiencing one or more long-COVID symptoms for at least 3 weeks following the onset of their initial symptoms. Minimum Duration: 8 weeks (approximately 2 months). Maximum duration: 52 weeks (one year). Average duration: 29 weeks (approximately 7 months). | To explore factors affecting mental health and well-being from the perspective of people with long-COVID | Around one in ten people who contract COVID-19 report persistent symptoms or ‘long COVID’. Impaired mental health and well-being is commonly reported, including anxiety, depression, and reduced quality of life. However, there is limited in-depth research exploring why mental health and well-being are affected in people experiencing long COVID | Semi-structured qualitative interviews | Reflexive thematic analysis | Five themes were identified across participant accounts regarding factors affecting mental health and well-being, including symptoms causing severe disruption to daily life, lack of service and treatment options, uncertainty of illness trajectories, experiences of care and understanding from others and changes to identity | High |
| Callan et al. (2022) | UK | 50 participants (42 were female and 32 white British). Most had never been hospitalised for COVID-19 | Participants were required to have experienced neurocognitive symptoms (e.g., "brain fog") following a COVID-19 infection. Minimum/average duration not stated. Participants’ symptoms were tracked for 10-12 months. | To explore the lived experience of ‘brain fog’— the wide variety of neurocognitive symptoms that can follow COVID-19 | Three key questions: (a) ‘what neurocognitive symptoms are experienced by adults with long COVID-19?’; (b) ‘what is the impact of these symptoms?’ and (c) ‘how do individuals deal with them?’. Study also sought to explore whether our understanding of cognitive processes/perceptions and the COVID-19 could inform potential causative explanations | Remotely held focus groups. Individuals were followed up by email 4–6 months later | Thematic analysis | 1. Naming the phenomenon. 2. Neurocognitive symptoms and their natural history. 3. Neurocognitive symptoms in the context of other long-COVID symptoms. 4. Psychosocial impact: guilt, shame and stigma. 5. Hypothesising mechanisms to inform self-management. 6. Navigating healthcare | High |
| Chasco et al. (2022) | USA | 15 patients of a Midwestern academic hospital’s post-COVID-19 clinic. The majority were females (66.7%) and received outpatient care (60%) during their acute illness | Participants were required to have persistent health concerns more than 3 months after a SARS-CoV-2 infection.  Range of duration: 6 to 20 months. Average duration not stated. | Study aimed to better understand the lived experiences of patients with post-acute sequelae of SARS-CoV-2 (PASC), focusing on the impact of cognitive complaints (“brain fog”) and fatigue on (1) daily activities, (2) work/employment, and (3) interpersonal relationships | Study aimed to better understand the lived experiences of patients with a particular focus on how patients describe cognitive complaints (“brain fog”) and fatigue, and the impact of these symptoms on daily activities, work/employment, and interpersonal relationships | Semi-structured telephone interviews | Thematic analysis | Impact on Daily Activities; Impact on Work/Employment; Impact on Interpersonal Relationships | High |
| Healthwatch Kingston (2022) | UK | 43 participants  (30 female, 7 male, 6 non-binary). Just over 80% were aged 25-64, nearly 8% aged 65-79, and nearly 8% over 80 years | Participants self-reported suffering from long-COVID. Range of Duration: 1 month to over 12 months. Average duration not stated. | The study aimed to gather Kingston residents’ views and experiences of NHS and social care services, to ensure commissioners, providers and other system leaders respond appropriately to the health and care needs of local people | Aim 1: Provision of accessible information informing of what to expect when recovering from Covid-19, self-management advice to aid recovery, and sources of support locally and nationally. Aim 2: Improve access to assessment and support for people from underserved and vulnerable groups, who are experiencing new or ongoing symptoms after (suspected/diagnosed) acute Covid-19. Aim 3: Continued development of integrated and coordinated services across the Kingston and Richmond system, that provide timely, holistic, and person-centred support. Aim 4: All people requiring a community assessment will be assessed with a holistic and person-centred approach and have access to multidisciplinary input in a format appropriate to their needs. Aim 5: People with long-COVID will be supported with a focus on returning to their usual activities of daily living, with this being measured alongside symptom management with use of the Kingston Hospital Post C-19 Holistic Questionnaire. Aim 6: Assessment and support for deterioration of mental health should be prioritised for all people experiencing long-COVID symptoms | Local online surveys | Not reported | The key findings concentrate on the respondents’ lived experience of long-COVID, including diagnosis, symptoms, effects on livelihoods, relationships, family, and friendships, and their views on clinical and community support needs (met and unmet), and mental health challenges. | High |
| Healthwatch North Central London (2022) | UK | 254 respondents (demographic data from were collected from 190).  More than half (51%) were aged 45 to 64. 79% were female, 20% were male and 1% were non-binary. 43% were from ethnic minorities | Participants self-reported suffering from long-COVID. Range of duration: 1-2 months to 12+ months. 73% reported that they had been living with long-covid for 6 months or more. Average duration not stated. | The study aimed to capture local people’s experiences of long-COVID in order to identify any gaps in current provisions; to support the better development of services and systems to help local people to manage their symptoms; to gather insight on local residents’ experiences of living with long-COVID | Adding to the knowledge required to develop a standardised treatment pathway from diagnosis to treatment and management of the condition | An online survey, one-to-one interviews, and community focus groups | Not reported | Findings: Impact on health; Impact on life; Experiences with the health care system; Moving forward | High |
| Healthwatch Wandsworth (2022) | UK | 47 participants (35 women, 12 men); nearly half were aged between 25 and 49 years. 26 were White British | Participants self-reported long-covid. The vast majority (42 out of 50) experienced long-covid symptoms for more than 6 months. Nearly half (22 out of 50) said they had had symptoms for more than a year. Average duration not stated. | The study aimed to understand what support is needed for people living with long-COVID or the long-term effects of another virus to inform decision makers about what people are experiencing and what further help may need to be put in place. | Recommendations to inform decision makers about people’s experiences and how they can design services and support to better meet people’s needs | Online survey | Not reported | Findings:  Symptoms; Impacts of the symptoms; Getting a diagnosis and experience of support; Current needs and possible improvements | Moderate |
| Humphreys et al. (2021) | UK (one participant from USA) | 18 people living with long COVID (9 men, 9 women; aged between 18–74 years; 10 white British) recruited via a UK-based research interest database for people with long COVID | Participants had to be adults who self-identified as recovering from COVID-19. They were not hospitalised and had experienced a recovery period lasting 3 weeks or more. Minimum/average duration not stated. | To explore the lived experience of long COVID with particular focus on the role of physical activity. Design Qualitative study using semi-structured interviews | The study explores the lived experience of people with long COVID, focusing on the role of physical activity, to inform the design and implementation of rehabilitation support | Semi-structured interviews | Reflexive thematic analysis | Theme 1 describes how participants struggled with drastically reduced physical function, compounded by the cognitive and psychological effects of long COVID.  Theme 2 highlights challenges associated with finding and interpreting advice about physical activity that was appropriately tailored.  Theme 3 describes individual approaches to managing symptoms including fatigue and ‘brain fog’ while trying to resume and maintain activities of daily living and other forms of exercise.  Theme 4 illustrates the battle with self-concept to accept reduced function (even temporarily) and the fear of permanent reduction in physical and cognitive ability | Moderate |
| Ireson et al. (2022) | UK | 66 participants (69% female, 31% male, 74% white British) | Participants were required to have experienced symptoms of long COVID. Minimum/average duration not stated. | The study aimed to explore the physical and epistemic challenges of living with long-COVID | A co-produced analysis of patient stories to further explore not only the physical nature of the condition but the epistemic challenges of the lived experience of long-COVID | 66 patient stories submitted online to covid19‐recovery.org at the beginning of the COVID-19 pandemic between April and September 2020 | Thematic analysis | Life changing:  End of normality  Rollercoaster symptoms  Uncertainty and fear with broader mental health impact  Validation:  Diagnosis  Access to care and services  Invisibility  Positive validation  Seeking alternatives:  Online peer support  Self-help  Exercise | High |
| Kennelly et al. (2023) | Canada | Lived experience advisory group consisting of six people living with Long COVID.  Final sample consisted of 47 people living with Long COVID, from diverse backgrounds. | Participants self-identified as  experiencing long COVID in accordance with the WHO definition (symptoms emerge or persist  three months after a confirmed or suspected case of SARS-CoV-2 infection and endure for  upwards of two months in the absence of an alternative explanation). Range of duration: 3-6 months to 13+ months. Average duration not stated. | The study aimed to gain insight into the mental health, quality of life and coping experiences of people living with long COVID throughout qualitative enquiry. | These themes illustrate the profound and enduring impact of long  COVID on daily life, to the extent that many participants experienced a sense of shattered identity due to the vast emotional, physical, and social impact of their experiences. Despite  these challenges, the extensive discussion of coping and help-seeking behaviour among participants  illustrates their commitment to reckoning with their new identity and advocating for an  improved experience for all people living with long COVID. | Web-based focus groups.  Separate focus groups were held for 24 individuals with pre-existing mental health conditions and 23 individuals without pre-existing mental health conditions | Data were analysed  using a codebook thematic analysis approach | The following five themes were recognised as central to the lived  experience of long COVID: The Emotional Landscape of Long COVID, New Limits to Daily  Functioning, Grief and Loss of Former Identity, Long COVID-related Stigmatization, and  Learning to Cope with Persisting Symptoms | High |
| Kingstone et al. (2020) | UK | 24 participants (5 males and 19 females). One participant was of mixed heritage; all other participants were white British or white other | Participants self-reported experiences of persistent symptoms following COVID-19 infection. Nearly all had been experiencing these symptoms since March or April 2020 (data were collected in July/August 2020). | To explore experiences of people with persisting symptoms following COVID-19 infection, and their views on primary care support received | Raise awareness among primary care professionals, and commissioners, of long-COVID and the range of symptoms people are experiencing | Semi-structured interviews | Thematic analysis | The main themes include: 1. the ‘hard and heavy work’ of enduring and managing symptoms and accessing care; 2. living with uncertainty, helplessness and fear, particularly over whether recovery is possible; 3.  the importance of finding the 'right' GP (understanding, empathy, and support needed); 4. recovery and rehabilitation: what would help? | Moderate |
| Knight et al. (2023) | UK | To participate, eligibility criteria mandated that individuals were aged over 18 years and had previously been infected with COVID-19. Overall, 47  adults (40 female), 6–11 months post-COVID-19 infection consented to participate.  Participants were recruited from the UK via social media and University webpages as part of a larger randomised  control trial (n = 281) investigating the use of inspiratory muscle training and were  invited to take part in both the wider intervention, and the qualitative interviews, until a target  of 50 pre-intervention interviews was achieved. | Participants self-reported suffering from long-COVID. All were 6–11 months post-COVID-19 infection. Average duration not stated. | The purpose of this study was to explore and present the day-to-day lived experiences of  adults in the United Kingdom (UK) who contracted COVID-19 during the early stages of the  pandemic, namely between February and May 2020. The primary focus was the secondary  socio-psychological effects and implications. | The vignettes outlined represent the all-too-often unheard voices of those who have experienced  COVID-19 and its aftereffects, especially those whose journey commenced long before we had any understanding of the trajectory it could take, or before strategies to facilitate recovery had started to be implemented.  From the point of initial  symptom development onwards, the vignettes depict how COVID-19 has affected everyday lives, focusing on the secondary non-biological socio-psychological effects and  implications. | Online semi-structured interviews.  Presented the lived experiences of individuals through the fusion of data created from  uniquely shared accounts. | Inductive thematic analysis | The vignettes highlight in participants’ own words: i) the potential negative implications of not addressing the psychological effects of COVID-19; ii) the lack of symptom  and recovery linearity; iii) the ongoing ‘lottery’ of access to healthcare services; and iv) the  highly variable, yet generally devastating, impacts that COVID-19 and consequent long-  COVID has had across multiple facets of daily living. | High |
| Ladds et al. (2020) | UK | 114 participants aged 27–73 years (80 female, 84 White British). Thirty-two were doctors and 19 other health professionals | The following inclusion criteria had to be met: symptoms developed between February and July  2020 following an acute illness consistent with COVID-19; symptoms continued beyond 3 weeks. Minimum/average duration not stated. | The study sought to document patients’ lived experience, including accessing and receiving healthcare and ideas for improving services | Suggested quality principles for a long Covid service – ensuring access to care, reducing burden of illness, taking clinical responsibility, and providing continuity of care, multi-disciplinary rehabilitation, evidence-based investigation and management, and further development of the knowledge base and clinical services | 55 individual interviews and 8 focus groups (n = 59) with people recruited from UK-based long-COVID patient support groups, social media and snowballing. The study restricted some focus groups to health professionals since they had already self-organised into online communities | Thematic analysis | Themes:  A serious, uncertain and confusing illness  Difficulty accessing and navigating services  Concerns about quality and safety of care  Emotional touch points | High |
| Ladds et al. (2021) | UK | 43 healthcare professionals  (35 female, 8 male, 36 white, 6 Asian, 1 black) | The following inclusion criteria had to be met: symptoms developed between February and July  2020 following an acute illness consistent with COVID-19; symptoms continued beyond 3 weeks. Minimum/average duration not stated. | The study sought to explore the experiences of healthcare workers with long-COVID to develop a set of quality standards and potential care pathway model for management of long-COVID | A set of co-designed quality standards, highlighting equity and ease of access, minimal patient care burden, clinical responsibility, a multidisciplinary and evidence-based approach, and patient involvement. These are applied to propose a potential care pathway model that could be adapted and translated to improve care of patients long COVID | Narrative interviews (11 participants) and focus groups (32 participants) | Thematic analysis | -Uncertainty  -use of mind lines  -support groups and communities of practice  -therapeutic relationships and roles  -professional identity and practice  -suggestions for service improvement | High |
| Leggat et al. (2023) | England and Wales | Participants aged over 18 years, who self-identified with Long Covid, were recruited from England and Wales.  18 participants (mean age = 44 years, SD = 13 years) | Participants self-reported long COVID. Range of duration: under 6 months to 19 months or more. Average duration not stated. | This study aimed to explore the range and influence of self-generated strategies used by people with Long Covid to navigate everyday life within the context of their own condition. | Forming part of the Long Covid Personalised Self-managemenT support co-design and EvaluatioN  (LISTEN) project, we conducted a qualitative study using narrative interviews with adults who were not hospitalised with Covid-19. | This study conducted a qualitative study using narrative interviews | Reflexive thematic analysis (TA) | Three themes were constructed from the analysis: 1)  *the landscape behind a Long Covid experience*, 2) *the everyday*  *experience,* and 3) *personal strategies to manage everyday life*. | High |
| Loft et al. (2022) | Denmark | 19 participants (15 female, 4 male) aged 25-63 | Participants had a clinical diagnosis of long-COVID. Duration ranged from 6 to 17 months. | The study explored the lived experiences of patients with long-term cognitive sequelae (LTCS) after recovering from COVID-19 | The focus was on gaining an in-depth understanding of patients’ lived experiences and overall significance of sequelae in their lives, while contributing to the emerging knowledge that can form future interventions | A qualitative design with in-depth interviews and an analysis inspired by Ricoeur’s interpretation theory | Phenomenological-hermeneutic approach | Five themes: 1) An unexpected journey: How LTCS Conquer Everyday Life; 2) Returning to Work and Surrendering to the Burden of LTCS; 3) A Lonesome Struggle: Managing LTCS Daily; 4) Socialising: An Energy Drain While Striving to Regain a Social Life; 5) A New Beginning: Navigating Hope and Despair on the Journey Towards a New Normal | High |
| McNabb et al. (2023) | USA | 24 adults with Long COVID, including 54% female and 45% male participants, with a median age of 46.5 years, predominantly White | Participants self-reported long-COVID. Minimum/average duration not stated. | The study explored the unmet supportive needs and experiences of people with Long COVID | Identification of three main areas affecting quality of life due to Long COVID: occupational and financial support, healthcare-related support, and social and emotional support | Qualitative sub-study; data were collected through in-depth interviews with participants enrolled in a larger study evaluating post-acute cardiovascular impacts of COVID-19 | Thematic content analysis, with focus on adaptations to life with Long COVID and unmet needs across different life spheres | Highlighted the persistent challenges in accessing appropriate care, employment difficulties, and inadequate governmental support programmes. Proposed a five-pronged policy approach to support persons with Long COVID, emphasizing improvements in public awareness, clinical care, workplace accommodations, socioeconomic benefits, and research | High |
| Moretti et al. (2022) | Italy | 17 women with long-COVID, aged 31 – 57 | Participants needed to have experienced long-COVID symptoms for at least three months following confirmed COVID-19 infection; have perceived a significant impact of long-COVID symptoms on quality of life; and have consulted multiple healthcare professionals in order to receive a diagnosis. Minimum/average duration not stated. | The study aimed to explore the illness experienced by people suffering from long-COVID in Italy, by analysing, in particular, the impact that the symptomatology has on the quality of life | A contribution to the development of flexible, person-centred interventions for people recovering and rehabilitating from long-COVID | Qualitative methodology with semi-structured interviews. Participants were recruited on a Facebook patient group and had been experiencing symptoms for at least three months following confirmed COVID-19 infection | Thematic analysis | Themes: a total change of life due to the symptomatology, loss of autonomy that affects social, family and professional life; social isolation, a sense of abandonment often increased by stigma, the difficulty of being believed and achieving diagnosis; difficulty in managing symptoms and accessing to care services; living with uncertainty caused by the lack of institutional, social, professional, familial and medical support | High |
| O’Brien et al. (2022) | Canada, Ireland, UK, and USA | 40 participants, (median age was 39 years; interquartile range: 32, 49); majority were women (63%), white (73%), and heterosexual (75%). | Participants self-identified as living with long-COVID, defined as signs and symptoms that develop during or following an infection consistent with COVID-19 which continue for 12 weeks or more and are not explained by an alternative diagnosis. 83% had been living with long-COVID for ≥1 year. | To describe the episodic nature of disability among adults living with long-COVID | A better understanding of the lived experiences and health-related challenges of people living with and affected by long-COVID to inform effective healthcare and rehabilitation approaches and interventions to enhance clinical practice, policy, and research | A community-engaged qualitative descriptive study involving online semi-structured interviews and participant visual illustrations.  Participants were asked to draw their health trajectory and conducted a group-based content analysis | Group-based qualitative analysis using content analytical techniques | Episodic terminology; Episodic disability as a continuum; Changes in episodic disability over time; Uncertainty living with long-COVID. | Moderate |
| Pearson et al. (2022) | UK | 28 submissions (no demographic data available) | Participants self-reported suffering from long-COVID. Minimum/average duration not stated. | The research aimed to collect the narratives of people living with long-COVID to better understand the lived experience of this condition | Creative expressions from participants with lived experience of long-COVID, as alternative ways of expressing embodied narratives, representing rich sources of meaning | Narrative methodology. Online repository where participants could submit their pieces of creative writing. Some pieces were in video or audio format | Thematic analysis | Five themes: Identity; social relationships; symptoms; interaction with healthcare systems; time | Low |
| Razai et al. (2021) | UK | 70 patients diagnosed with COVID were randomly selected from two primary care practices.  Mean age was 49, age range was 19-82, 27 (66%) were female | Participants were patients with suspected and/or laboratory-confirmed COVID-19. All participants were 4 weeks post-diagnosis. | The study aimed to explore patients’ acute and post-acute long-COVID symptoms, their experiences of community services and their recommendations for improving these services | Exploration of patients’ acute and post-acute long-COVID symptoms, their experiences of community services and their recommendations for improving these services | Interviews using semi-structured questionnaire | Thematic analysis | Four themes: living with fear and uncertainty; the impact of long-COVID on patients’ lives; experiences of accessing GP care; recommendations to improve services to support recovery from long-COVID | Moderate |
| Rofail et al. (2023) | USA | 41 adult patients with long COVID-19, mostly female (85.4%) and White (73.2%). Patients were recruited through clinical trials and external agencies | Participants had to have experienced long COVID-19 symptoms for at least 180 days (6 months) following a positive SARS-CoV-2 PCR test.  The symptoms could not be explained by an alternative diagnosis. Average duration: 12.2 months. | The study explores the lived experiences of long COVID-19 patients, aiming to develop a conceptual model of the symptoms and their impacts on daily lives | Development of a conceptual model categorizing symptoms (such as neurocognitive and respiratory symptoms) and impacts on activities of daily living, professional life, and emotional well-being | Qualitative study consisting of a comprehensive literature review and in-depth interviews with patients and clinicians | Thematic analysis was used to identify spontaneously mentioned concepts, leading to the development of a conceptual model | Findings include a range of symptoms that affect patients' physical and mental health and extensive impacts on daily living, work, and emotional states. The study emphasized the complexity and persistence of neurocognitive symptoms | High |
| Rushforth et al. (2021) | UK | 114 people with long-COVID. Median age was 48 years (range 27-73). 84 were White British. Gender not reported | Participants self-reported suffering from long-COVID Minimum/average duration not stated. | The study aimed to explore long-COVID’s rapid emergence and unique status among illnesses, using a dataset of narrative interviews and focus groups with people with long-COVID | Addressing the following questions: - What kinds of stories did people with long-COVID tell – and what did they seek to achieve by telling them? - How did storytelling inform and inspire new individual and collective identities and collective action? - How did the absence (in most cases) of the storyteller’s clinician as therapeutic witness influence both individual narratives and the collective response? | Individual qualitative interviews (55 participants) and focus groups (59 participants) | Thematic analysis | Online communities;  a strange kind of trouble;  disrupted chronology and the cruelty of hope;  a doubting audience; frustrated becoming a test character;  the healthcare system as a lottery;  clinicians in the narratives: present and absent witnesses;  the online community: collective witnessing and knowledge sharing; taking collective action | High |
| Russell et al. (2022) | USA | 20 people living with long-COVID(16 women, 3 men, 1 non-binary) aged between 18 and 55+ years. 85% were White non-Hispanic | Participants self-identified as suffering from long-COVID. Average duration: 232 days. Minimum duration not stated. | The study aimed to explore the subjective experiences of 20 persons with long-COVID recruited from five online communities. Their understandings of illness and associated implications for social relationships with family and friends, healthcare professionals, and online community members were explored | A detailed account of the subjective experiences of U.S. adults with long-COVID, including their understandings of illness and relationships with healthcare professionals and online communities | Semi-structured interviews | Thematic analysis | Three themes: (1) complex and unpredictable illness experienced amid an evolving understanding of the pandemic; (2) frustration, dismissal, and gaslighting in healthcare interactions; and (3) validation and support from online communities | High |
| Samper-Pardo et al. (2023) | Spain | 35 patients (25 female, 10 male) aged 20 to 60+ years | Participants had to have been diagnosed with long-COVID by a general  practitioner or specialised doctor, and to have tested positive for COVID-19. Average duration: 15 months. Minimum duration not stated. | The study aimed to increase understanding of the emotional well-being of people diagnosed with long-COVID | A deepening of understanding of the emotional well-being of people diagnosed with long-COVID, as well as their social support and experiences of discrimination and social stigma | Qualitative design. 17 participants were interviewed individually and 18 took part in focus groups | Inductive thematic content analyses | 3 main themes: 1) emotional well-being; 2) social support networks; and 3) experiences of discrimination and perceived social stigma | High |
| Santiago-Rodriguez et al. (2021) | USA | 24 participants. Median age was 49, and most were men (62.5%) and white (66.7%) | Participants were individuals with previously documented SARS-CoV-2 infection who had entered the “recovery phase” from COVID-19. Minimum/average duration not stated. | The study characterised the variability in the acute illness experience and ongoing recovery process from participants in a COVID-19 recovery cohort study in Northern California in 2020 | The study informs the emerging field of long-COVID research and shows a need to provide information and continuous support to persons with post-acute sequelae to ensure they feel secure along the path to recovery | Semi-structured in-depth interviews | Thematic analysis | (1) across symptom profiles and severity, experiencing COVID-19 was associated with psychological distress; (2) symptomatic infection carried uncertainty in symptom presentation and ongoing recovery (e.g., long-COVID); and (3) health information-seeking behaviour was facilitated by access to medical care and uncertainty with the recovery process | High |
| Schiavi et al. (2022) | Italy | 56 participants (34 male, 22 female) | Participants were adult symptomatic individuals hospitalised for  COVID-19 during the first peak of the pandemic and discharged from the hospitals of the Azienda USL-IRCCS  of Reggio Emilia (Italy). Minimum duration: 3 months. Average duration not stated. | The study investigated the experience of individuals who had been hospitalised for COVID-19, focusing on those needs and difficulties they perceived as most urgent | The experience as narrated by the participants in this study highlights the sense of isolation and psychological distress. These phenomena may trigger a  vicious circle, but the participants also reported adaptation processes that allowed them to gradually return to their life course | Naturalistic qualitative study | Deductive thematic analysis | Six themes:  persistent symptoms, feelings of isolation,  fear and stigma, emotional distress, fatalistic attitude (“goodnight”),  and return to their (adapted) life course | High |
| Skilbeck et al. (2023) | UK | 18 adults referred to a primary care integrated psychology service, living with long COVID. Participants ranged in age, gender, and ethnicity | Participants self-reported to have experienced long-COVID. Participants had long-COVID for a range of 10 to 24 months. Average duration not stated. | The study aimed to capture the phenomenological lived experiences of individuals with long COVID, emphasising the biopsychosocial impacts of the condition | The study highlighted the significant psychosocial challenges faced by individuals with long COVID, including the struggle with identity, the unpredictability of symptoms, and the effort to regain a sense of control and normalcy in their lives | Qualitative research using semi-structured interviews analysed through Interpretive Phenomenological Analysis (IPA) | Thematic analysis revealed complex themes around the uncertainty and chronic nature of long COVID, impacting mental and physical health, and strategies developed by individuals to manage their symptoms and regain control | Themes included dealing with an unknown chronic illness, living with uncertainty, regaining control, and moving forward. Participants expressed varied experiences but commonly reported significant disruptions to their personal and professional lives, alongside strategies to manage and cope with their condition | High |
| Spence et al. (2023) | USA | 20 working-aged adults in the USA who self-identified as "long-haulers," experiencing long-term effects post-COVID-19 | Participants had to have long-term symptoms and effects from COVID-19. They must have had at least one consultation with a healthcare professional about their COVID-19 symptoms. Range of duration: 96 days to 377 days. Average Duration: 232 days (approximately 7.7 months). | Investigated the biographical disruptions and identity changes experienced by individuals living with long COVID | The study detailed the three stages of identity disruption due to long COVID: realizing disruptions, identity and role challenges, and reconciling illness with identity amid ongoing uncertainty about their health prognosis | Qualitative study using in-depth interviews | Thematic analysis focusing on identity disruptions as a central theme, detailing the profound effects on personal identity and social roles due to long COVID | Describes the significant and ongoing impact of long COVID on individuals' professional lives, social identities, and personal expectations. Highlights the importance of social support networks, particularly online communities, in helping individuals cope with the uncertain prognosis and identity changes | High |
| Sunkersing et al. (2024) | UK | 21 adults with Long COVID, 18 female and 3 male, various age ranges, predominantly white, from six different NHS post-COVID-19 services. Additionally, 15 healthcare professionals were interviewed. | Participants were required to have a confirmed or clinically diagnosed case of long COVID.  Minimum/average duration not provided. | Investigated current care for people with Long COVID in England, exploring patient and healthcare professional perspectives | Diverse experiences in care access, multifaceted impacts of Long COVID, and varying quality of post-COVID-19 services. Highlights the need for effective communication, specialised expertise, and comprehensive support systems | Qualitative; In-depth, semi-structured interviews with 21 people living with Long COVID and 15 healthcare professionals | Thematic analysis revealing complexities of managing Long COVID, including the physical, social, mental, and environmental dimensions | Reports on barriers in accessing primary care and variable experiences with healthcare professionals. Notes positive impacts of peer support and the value of specialised post-COVID-19 services | High |
| Taylor et al. (2021) | UK | 13 doctors – 11 females, 11 White British | Participants were doctors experiencing persistent symptoms following COVID-19 or suspected COVID-19  infection. All participants had developed symptoms in March or April 2020 (data were collected in July/August 2020). | This paper reports the experiences of doctors with long-COVID | An exploration of experiences of people with persisting symptoms following suspected or confirmed COVID-19 infection in March or April 2020, which became known as ‘long-COVID’ during the course of their illness | Qualitative interviews | Inductive thematic approach | Themes:  making sense of symptoms;  feeling let down;  using medical knowledge and connections;  wanting to help and be helped;  combining patient and professional identity | Moderate |
| Wang et al. (2022) | China | 64 answers – text questions on app (no demographic data available) | Self-narratives of long COVID patients on the Zhihu App were examined. Minimum/average duration not stated. | The study coded the self-produced texts of long-COVID patients on the largest online Q&A community in China, Zhihu APP, in an attempt to explore the illness experiences of long-COVID patients in China and to understand how they adapt to their illness and reconstruct their lives | The study aimed to add to the information about the emerging field of long-COVID and echo the existing research | Self-narratives of long-COVID patients on the Zhihu App were analysed | Grounded theory | Two themes: The Disordered Body and Life, and Reconstructing Self and Life | Moderate |
| Wurz et al. (2022) | Canada, UK, and USA | 169 participants who primarily self-identified as women (88.2%), aged 40–49 (33.1%), who had been experiencing long-COVID symptoms for≥6 months (74%) | Participants self-identified as currently experiencing long-term symptoms due to  COVID-19 (at least 4 weeks since the acute illness or positive  COVID-19 test, with symptoms not pre-dating the acute illness); and having tested positive for COVID-19, or  with probable infection (based on an illness mimicking the  acute phase of COVID-19, having close contact with a confirmed  case, or being linked with an outbreak), in line with the clinical case definition post-COVID-19 condition.  Most (58.6%) reported managing long COVID symptoms for more than 10 months. Range of duration: 1-2 months to 10+ months. | The study aimed to better understand and explore individuals' experiences with long-COVID and commonly reported symptoms, using qualitative data collected from open-ended survey responses | A better understanding and exploration of individuals' experiences with long-COVID and commonly reported symptoms using qualitative data collected from open-ended survey items | The qualitative data reported were collected as part of a larger observational study using an online survey | Reflective thematic analysis | Four themes: 1. Long-COVID symptoms are numerous and wearing; 2. The effects of long COVID are pervasive; 3. Physical activity is difficult and, in some cases, not possible;  and (4) Asking for help when few are listening, and little is working | Moderate |

Appendix S1. Systematic Review Protocol

# **T**[**he psychosoci**](http://www.nihr.ac.uk/)**al aspects of the lived experience of long-covid: a systematic review and meta-ethnography**

*Stephanie Kilinc, Judith Eberhardt, Sam Rowlands, Rachel Batchelor*

Review methods were amended after registration. Please see the revision notes and previous versions for detail.

**Citation**

Stephanie Kilinc, Judith Eberhardt, Sam Rowlands, Rachel Batchelor. The psychosocial aspects of the lived experience of long-covid: a systematic review and meta-ethnography. PROSPERO 2022 CRD42022343091 Available from: <https://www.crd.york.ac.uk/prospero/display_record.php?ID=CRD42022343091>

**Review question**

The psychosocial impact of living with long-covid has received limited attention (Maxwell, 2020) and should not be overlooked in favour of purely medical and rehabilitation approaches to symptoms (Lyons et al., 2020). This review aims to synthesise current evidence to identify and explore key themes which illustrate the psychosocial aspects of the lived experience of long-covid; looking beyond the impact of long-covid symptoms and experiences of healthcare, to the broader psychosocial impact of living with long-covid.

The research question is:

What are the psychosocial aspects of the lived experience of long covid?

Lyons, D. et al. (2020). Fallout from the COVID-19 pandemic – should we prepare for a tsunami of post viral depression? Irish Journal of Psychological Medicine, 1-6. doi:10.1017/ipm.2020.40

Maxwell, E. (2020). Living with Covid19. A dynamic review of the evidence around ongoing Covid19 symptoms (often called Long Covid). NIHR: https://evidence.nihr.ac.uk/themedreview/living-with-covid19/

**Searches**

Sources:

PsycINFO, MEDLINE, AMED, APA, CINAHL, Psychology and Behavioural Sciences Collection.

Grey literature: reports from organisations such as NHS, Health Foundation Kings Fund, NIHR, Royal Society, Tony Blair Institute for Global Change

In order to gather data for the qualitative synthesis, literature searches will be carried out using the SPIDER framework

Sample: People living with long-covid

Phenomenon of Interest: Psychosocial aspects of living with long-covid Design: Any design which uses participant quotes

Evaluation: Studies related to the lived experience of people with long-covid, excluding lived experience of

their family or carers

Research type: Qualitative, including mixed methods studies which contain sufficient qualitative data, excluding qualitative evaluations of specific interventions

Additional search strategy information can be found in the attached PDF document (link provided below).

**Types of study to be included** [1 change]

The review will include primary research studies and long-covid relevant organisation reports which have gathered qualitative data from adults living with long-covid which focuses on analysis of the psychosocial aspects of their lived experience of long-covid. If the sample includes non long-covid sufferers, the data should be clearly attributable to long-covid sufferers. Mixed methods studies which contain sufficient qualitative data will be included in the review. The review will include papers and reports from worldwide, provided they are translated into English.

Excluded: Magazines, books, dissertations/theses, conference abstracts and posters, literature not reporting first person experiences of long-covid, quantitative studies, articles where the full text cannot be retrieved, papers/reports with children, qualitative evaluations of specific interventions, papers/reports which are not reporting primary research.

**Condition or domain being studied** [1 change]

Long-covid, as self-reported by participants. Typically,

**Participants/population** [1 change]

Inclusion: adults (18+ years) who self-report as living with long-covid.

Exclusion: lived experience of family members or carers of people living with long-covid, papers/reports focused on children and adolescents, under the age of 18.

**Intervention(s), exposure(s)**

Inclusion: any qualitative methods of data collection which collect data from people living with long-covid and focus on their lived experience of long-covid. Mixed methods studies will be included if they contain sufficient qualitative data to warrant inclusion.

Exclusion: qualitative evaluations of specific interventions.

**Comparator(s)/control**

Not applicable.

**Context**

The review will include papers and reports from worldwide, provided they are translated into English.

**Main outcome(s)**

To explore the psychosocial aspects of the lived experience of long-covid.

**Additional outcome(s)**

Exploring the lived experience of long-covid in a broader sense; looking beyond the impact of long-covid symptoms and experiences of healthcare, to the broader psychosocial impact of living with long-covid.

**Data extraction (selection and coding)**

PRISMA will be used to ensure rigour during the data extraction process. There are four reviewers included throughout the process. The first reviewer (SK) will conduct the searches and export the search results in to Zotero where duplicates will be removed. The first reviewer will screen all papers/reports by title and abstract and the second reviewer (SR) will blind screen 20% of the papers/reports by following the inclusion and exclusion criteria stated in the protocol. A meeting will be held between both reviewers to discuss any discrepancies and reach a consensus. The first will then retrieve the full texts of the selected papers/reports

and they will be screened for relevance by both the first reviewer and the third reviewer (JE) independently. Consensus over inclusion will be reached via a meeting between both reviewers.

Data extraction will include: title; author; year published; country of origin; study aim; method of data collection; data analysis; study population characteristics. An inclusive approach to data extraction will be utilised, as advocated for meta-ethnography by Noblit and Hare (1988).

The first reviewer will appraise the quality of the studies using the Critical Appraisal Skills Programme (CASP) tool and reviewer four (RB) will check 20% for agreement. Any disagreement in CAPS ratings will be discussed in a meeting between both reviewers and a consensus reached. No study will be excluded as a result of using the CASP tool, though it will be used to provide context to the review. The first reviewer will then conduct a meta-ethnography, following Noblit and Hare’s (1988) guidelines.

Noblit, G. W. and Hare, R. D. (1988) Meta-Ethnography: Synthesizing Qualitative Studies. Sage Publications, Inc.

**Risk of bias (quality) assessment**

The first reviewer will appraise the quality of the studies using the Critical Appraisal Skills Programme (CASP) tool and reviewer four (RB) will check 20% for agreement. Any disagreement in CASP ratings will be discussed in a meeting between both reviewers and a consensus reached.

Since meta-ethnography is an interpretative process, the first reviewer will conduct the analysis and reviewers two and four will review each step in the process to ensure rigour. All three reviewers involved in this process will engage with reflexivity by completing their own reflexive diaries throughout the analysis process.

**Strategy for data synthesis**

Data synthesis will follow the seven steps of meta-ethnography outlined by Nobblit and Hare (1988). Firstly, a list of metaphors or ideas from the findings of each study will be produced and reoccurring concepts identified across the studies. Secondly, the findings from each study will be compared against the concepts identified in the previous stage to refine the concepts further. A list of the articles associated with each concept will then be produced before finally synthesising the findings into categories describing the psychosocial aspects of the lived experience of long-covid.

**Analysis of subgroups or subsets**

None planned.

**Contact details for further information**

Stephanie Kilinc [s.kilinc@tees.ac.uk](mailto:s.kilinc@tees.ac.uk)

**Organisational affiliation of the review**

Teesside University [www.tees.ac.uk](https://www.crd.york.ac.uk/prospero/www.tees.ac.uk)

**Review team members and their organisational affiliations** [1 change]

Dr Stephanie Kilinc. Teesside University Dr Judith Eberhardt. Teesside University Mr Sam Rowlands. Teesside University

Miss Rachel Batchelor. Sussex Partnership NHS Foundation Trust

**Type and method of review**

Epidemiologic, Systematic review, Other

**Anticipated or actual start date**

30 June 2022

**Anticipated completion date** [2 changes]

25 March 2023

**Funding sources/sponsors**

None

**Conflicts of interest**

**Language**

English

**Country**

England

**Stage of review** [1 change]

Review Completed not published

**Subject index terms status**

Subject indexing assigned by CRD

**Subject index terms**

Adult; COVID-19; Humans; Mental Disorders: Mental Health; Psychosocial Functioning; SARS-CoV-2; Survivors; Syndrome

| **Date of registration in PROSPERO** |  | |
| --- | --- | --- |
| 29 June 2022 |  |  |
| **Date of first submission**  29 June 2022 |  |  |
| **Stage of review at time of this submission** [2 changes] |  |  |
| **Stage** | **Started** | **Completed** |
| Preliminary searches | Yes | Yes |
| Piloting of the study selection process | Yes | Yes |
| Formal screening of search results against eligibility criteria | Yes | Yes |
| Data extraction | Yes | Yes |
| Risk of bias (quality) assessment | Yes | Yes |
| Data analysis | Yes | Yes |
| **Revision note** |  |  |

Review is complete and paper submitted to a journal.

*The record owner confirms that the information they have supplied for this submission is accurate and complete and they understand that deliberate provision of inaccurate information or omission of data may be construed as scientific misconduct.*

*The record owner confirms that they will update the status of the review when it is completed and will add publication details in due course.*

**Versions**

[29 June 2022](https://www.crd.york.ac.uk/prospero/display_record.php?RecordID=343091&VersionID=1747177)

[19 July 2022](https://www.crd.york.ac.uk/prospero/display_record.php?RecordID=343091&VersionID=1759564)

[01 October 2022](https://www.crd.york.ac.uk/prospero/display_record.php?RecordID=343091&VersionID=1807770)

[21 October 2022](https://www.crd.york.ac.uk/prospero/display_record.php?RecordID=343091&VersionID=1820892)

[19 June 2023](https://www.crd.york.ac.uk/prospero/display_record.php?RecordID=343091&VersionID=1994435)
